# Progenitor cell fate, SOXC and WNT

**DOI:** 10.18632/oncotarget.5237

**Published:** 2015-08-22

**Authors:** Pallavi Bhattaram, Kenji Kato, Véronique Lefebvre

**Affiliations:** Cellular and Molecular Medicine, Cleveland Clinic Lerner Research Institute, Cleveland, OH, USA

**Keywords:** SOXC, WNT signaling, cell fate and differentiation, skeletogenesis, Chromosome Section

SOX4, SOX11 and SOX12 form the group C in the family of SOX transcription factors [1]. Overall, the SOX family has prominent roles in determining the lineage fate and differentiation of virtually all cell types in humans and animals, each member sponsoring a discrete set of lineages. Most SOX family members achieve their functions through transcriptional actions, and complement them with non-transcriptional interactions. For instance, SOX9 is necessary for and directly activates most genes defining the chondrocyte early program and it also binds to β-catenin to trigger the protein degradation and thereby dampens the anti-chondrogenic canonical WNT signaling. Recent studies, including our own, have started to reveal unique SOXC contributions to this pivotal SOX family mission.

The *SoxC* genes widely overlap in expression from development onwards and they are most active in progenitor/stem cells [2–4]. They encode proteins with almost identical DNA-binding domains, but SOX11 and SOX4 exhibit a much stronger transactivation domain than SOX12. Often working in a redundant or complementary manner, SOX4 and SOX11 are critically needed for progenitor cell survival in numerous processes, e.g., cardiac formation, neurogenesis, skeletogenesis and tumorigenesis. In contrast, SOX12 is only a modest rheostat that adds to or interferes with the activities of SOX4 and SOX11 [3–6]. SOX4 and SOX11 were shown to directly control differentiation genes in neuronal cells, but were found dispensable for lineage-specific gene expression in other cell types. Instead, they were shown to control genes encoding central regulators of a number of important molecular networks, such as the p53, PI3K/AKT, Hedgehog, HIPPO, NOTCH, and TGFβ pathways [4, 7]. Our recent studies on the importance of SOXC proteins in skeletogenesis have added two more pathways to this list.

Skeletogenesis starts in the embryo with the aggregation of mesenchymal progenitors. Cells located in the core of most condensations undergo chondrogenesis, driven by SOX9, whereas peripheral cells relinquish the chondrogenic fate, led by canonical WNT/β-catenin signaling. While peripheral cells generate perichondrium/periosteum and articular joints, chondrocytes establish growth plates, structures that steer skeleton elongation. They actively proliferate and pile up in longitudinal columns, then undergo hypertrophic maturation in a staggered manner, and ultimately die or help replace cartilage by bone. *SoxC* expression is robust in mesenchymal progenitors, remains strong in presumptive perichondrium/periosteum and joint cells, and quickly weakens in differentiated chondrocytes [5, 6]. Inactivation of either *SoxC* gene has limited consequences on skeletogenesis, but co-inactivation of the three genes causes severe skeletal dysplasia. Many progenitor cells die before precartilaginous condensation [4, 5]. Surviving ones undergo chondrogenesis in cartilage primordia, as expected, but also in presumptive perichondrium and joints, leading to fusion of the hundreds of cartilage primordia that normally constitute the developing skeleton. In addition, growth plates fail to form, resulting in drastic dwarfism and lack of ossification. Conditional mouse mutant experiments have strongly suggested that SOXC proteins trigger growth plate development mainly through actions in perichondrocytes. *In vivo* and *in vitro* experiments demonstrated that SOXC proteins bind β-catenin, but unlike SOX9, they block β-catenin degradation and thereby powerfully amplify canonical WNT signaling. This non-transcriptional activity effectively downregulates *Sox9* expression in peripheral cells, and hence secures the non-chondrocytic fates of these cells. This allows proper delineation and articulation of skeletal elements. In chondrocytes, *Sox9* expression dominates over *SoxC* expression, keeping canonical WNT signaling in check and permitting the antagonistic non-canonical WNT pathway to induce growth plate development. This pathway is primarily led by WNT5A, produced weakly by chondrocytes, but robustly by perichondrocytes. We showed that SOXC proteins are necessary for expression of *Wnt5a* and other pathway components in both cell types, likely through direct transcriptional actions [7]. This explains that SOXC proteins act on growth plate chondrocytes mainly non-cell-autonomously. SOXC proteins thus participate uniquely in skeletogenesis. By significantly promoting canonical and non-canonical WNT signaling, they help ensure skeletogenic cell survival, fate choice and functions, including signaling interactions with differentiated neighbors.

As canonical and non-canonical WNT pathways are widely active and as SOXC proteins are widely expressed, we anticipate that SOXC proteins may promote these pathways in many processes beside skeletogenesis. Moreover, as SOXC proteins control many other pathways in various cell types and as skeletogenesis involves many pathways, SOXC proteins may orchestrate WNT and other pathways during skeletogenesis. Last but not least, considering all data currently available, we like to propose that SOXC proteins may control progenitor/stem cell fate and actions in many lineages using mechanisms largely distinct from those used by other SOX proteins. Instead of primarily regulating cell type-specific genes, SOXC proteins may mainly act to empower non-cell-type-specific signaling pathways. This mode of action may contribute to confer on progenitor/stem cells their characteristic, highly versatile ability to promptly adjust to extrinsic cues.
